# Interaction between NOD2 and CARD9 involves the NOD2 NACHT and the linker region between the NOD2 CARDs and NACHT domain

**DOI:** 10.1016/j.febslet.2014.06.035

**Published:** 2014-08-25

**Authors:** Rhiannon Parkhouse, Joseph P. Boyle, Sophie Mayle, Kovilen Sawmynaden, Katrin Rittinger, Tom P. Monie

**Affiliations:** a Department of Biochemistry, University of Cambridge, Cambridge, UK; b Division of Molecular Structure, MRC-National Institute for Medical Research, London, UK; c Department of Veterinary Medicine, University of Cambridge, Cambridge, UK

**Keywords:** NOD, nucleotide oligomerisation domain, NLR, nucleotide-binding leucine-rich repeat containing receptor, NF-κB, nuclear factor kappa B, CARD, caspase activation and recruitment domain, RIP2, receptor interacting protein 2, SNP, single nucleotide polymorphism, MBP, maltose binding protein, Nucleotide-binding leucine-rich repeat containing receptor, Crohn’s Disease, Caspase activation and recruitment domain, Stress kinase pathway, Innate immunity, Signal transduction

## Abstract

NOD2 activation by muramyl dipeptide causes a proinflammatory immune response in which the adaptor protein CARD9 works synergistically with NOD2 to drive p38 and c-Jun N-terminal kinase (JNK) signalling. To date the nature of the interaction between NOD2 and CARD9 remains undetermined. Here we show that this interaction is not mediated by the CARDs of NOD2 and CARD9 as previously suggested, but that NOD2 possesses two interaction sites for CARD9; one in the CARD–NACHT linker and one in the NACHT itself.

## Highlights

• We have studied the interaction between NOD2 and CARD9.
• The NACHT domain and CARD–NACHT linker of NOD2 are crucial for the interaction.
• The CARD domains of NOD2 and CARD9 do not directly interact.

## Structured summary of protein interactions

**NOD2** physically interacts with **CARD9** by anti tag coimmunoprecipitation (View interaction)

## 1 Introduction

The cytoplasmic proteins NOD (nucleotide-binding oligomerisation domain containing) 1 and NOD2 are members of the NLR (nucleotide-binding, leucine-rich repeat containing receptor) family of pattern recognition receptors. They act as immune sentinels and play an important role in combating bacterial infection and maintaining cellular homeostasis [1]. NOD1 and NOD2 recognise fragments of bacterial peptidoglycan via their C-terminal leucine rich repeats [2], [3], [4], [5]. Activation causes conformational change and relocalisation to the plasma membrane [6], [7], [8], [9]. NF-κB (nuclear factor kappa B)-mediated pro-inflammatory signalling pathways are activated following interaction between the CARDs (caspase activation and recruitment domain) of NOD1/2 with their adaptor protein RIP2 (receptor interacting protein 2). Understanding how NOD1/2 signalling is regulated is important for the future development of therapeutic treatments targeting inflammatory disorders such as Crohn’s Disease.

CARD9 is an important adaptor molecule in the innate immune response. CARD9 is predominantly associated with NF-κB signalling pathways following stimulation of C-type lectin receptors like DECTIN-1 [10], [11]. Receptor ligation upregulates Spleen tyrosine kinase (SYK) and activates Protein Kinase C delta which phosphorylates Thr231 in CARD9 [12]. This causes formation of a CARD9/B Cell lymphoma 10 (Bcl-10)/Mucosa-associated lymphoid tissue lymphoma translocation protein 1 (Malt1) ‘signalosome’ which activates NF-κB [10], [13]. CARD9 is also involved in NF-κB signalling downstream of the Retinoic acid-inducible gene I receptor (RIG-1) family [14]. The biological function of CARD9 is conserved between mice and humans. Human CARD9 is able to restore signalling in murine *Card9* knock-out cells [15] and both inactive human *CARD9* mutant cells and murine *Card9* knockout cells display a defective response to β-glucan stimulation [15], [16]

Recently, Hsu and colleagues demonstrated in mice that *CARD9* is required for the synergistic activation of p38 and JNK (c-Jun N-terminal kinase) following stimulation of NOD2 by either muramyl dipeptide or *Listeria monocytogenes* [17]*.* The association between NOD2 and CARD9 was enhanced by the presence of RIP2 in both over-expression and endogenous systems [17]. The relationship between CARD9 and NOD2 is particularly intriguing as the genes for both these proteins contain polymorphisms influencing susceptibility to Crohn’s Disease in humans [18], [19].

Multiprotein complexes play a key role in innate immune signalling. Complexes such as the inflammasome and Myddosome are formed through interactions between members of the death domain superfamily [20], which includes CARDs. NOD2 and CARD9 have two and one N-terminal CARDs respectively. We have used cell-based immunoprecipitation and co-purification of overexpressed recombinant protein to study the molecular details of the interaction between NOD2 and CARD9. Unexpectedly, we did not find any evidence for an interaction between the CARDs of NOD2 and CARD9 as previously suggested. Instead, we show that the region in NOD2 responsible for the interaction with CARD9 involves the NACHT domain and the preceding linker to the CARDs.

## 2 Materials and methods

### 2.1 Plasmids

Full length murine CARD9 (GENBANK: NP_001032836.1) with a C-terminal V5-His epitope tag in the pEF6 expression vector (pEF6-mCARD9-V5) was a kind gift from David Underhill [11]. Full-length human NOD1 (GENBANK: AAD28350.1) and NOD2 (GENBANK: AAG33677.1) with an N-terminal FLAG tag in a pCMV backbone (pCMV-FLAG-NOD1 and pCMV-FLAG-NOD2) were kindly provided by Thomas Kufer [21]. Single nucleotide polymorphisms (SNP) across the NACHT domain were identified in the NCBI SNP database and cloned into full-length pCMV-FLAG-NOD2 using site-directed mutagenesis. N-terminal and C-terminal NOD2 constructs were also generated by site-directed mutagenesis. GB1-RIP2–CARD (human) and the tandem human NOD2 CARD construct used for NMR chemical shifts have been described previously [22]. The CARD of Card9 (residues 6-100), the tandem CARDs of NOD2 (residues 28-215) and the RIP2 CARD (residues 432-534) were amplified by PCR and inserted, using Gateway® cloning, into the expression plasmid pDEST-HisMBP [23]. In addition to the N-terminal His6-MBP (maltose binding protein) fusion each construct included a C-terminal FLAG tag to further aid expression and stability. The CARD of murine CARD9 was also inserted into pDEST-17 to generate an N-terminally 6His tagged construct.

### 2.2 Immunoprecipitation

HEK 293T cells were maintained in DMEM (Sigma) supplemented with 10% FCS, 100 μg/ml Penicillin/Streptomycin and 2 mM l-glutamine at 37 °C and 5% CO_2_. Cells were seeded in 6 well plates and transfected using jetPEI (Polyplus Transfection) with 1 μg/well of full-length, mutated, or truncated pCMV-FLAG-NOD2, or full-length pCMV-FLAG-NOD1, and 1 μg pEF6-mCARD9-V5. After 24 h cells were washed twice in 1 × PBS and lysed in 300 μl RIPA buffer (50 mM Tris–HCl pH 7.6, 150 mM NaCl, 0.25% Triton X-100, 0.1% SDS, 0.5% sodium deoxycholate) supplemented with 1 × Protease Inhibitor Cocktail set V (Calbiochem) and 7.5 units of Benzonase nuclease (Sigma) per well. Lysates were incubated on ice for 10 min with shaking and clarified by centrifugation (16 000×*g*; 2 min; 20 °C). Cells were lysed and incubated with Protein G coated magnetic Dynabeads (Life Technologies) coupled to mouse anti-FLAG antibody (Sigma). Immunoprecipitated proteins and inputs were detected by western blot analysis using: rabbit anti-flag (Sigma), mouse anti-V5 (Abcam) and mouse anti-GAPDH (Abcam) primary antibodies and goat anti-rabbit (Abcam) or goat anti-mouse (Sigma) secondary antibodies.

### 2.3 Recombinant protein expression and co-sonication pull-down assays

Recombinant proteins were expressed in 10 ml cultures of Rosetta2 *Escherichia coli* for 16 hr at 22 °C except for GB1-RIP2–CARD which used 4 h at 37 °C. Cultures were pelleted, frozen overnight and resuspended in standard lysis buffer (100 mM NaCl, 25 mM sodium phosphate, 20 mM imidazole, 5 mM β-mercaptoethanol, 0.1 % Triton-X100). Appropriate samples were combined and sonicated on ice. Insoluble debris was removed by centrifugation (16 000×*g*, 4 °C, 10 min) and proteins purified using amylose affinity chromatography. Eluted samples were visualised by Coomassie Brilliant Blue staining.

### 2.4 NMR chemical shift assays

Samples of unlabelled murine CARD9 CARD and ^15^N-labelled human NOD2 (28–218) were buffer exchanged overnight into 20 mM sodium phosphate (pH 7.1), 100 mM NaCl and 5 mM DTT. 1D (^1^H) and 2D (^1^H/^15^N) HSQC NMR spectra were recorded on a Bruker Avance spectrometer at 600 MHz proton frequencies and processed using an associated software package. All spectra were recorded at 180 μM sample concentration (with 10% added D_2_O) and at 298 K.

### 2.5 Homology modelling and bioinformatics

Residues 217–820, corresponding to residues encoded by the large, central exon of human *NOD2*, were submitted to the PHYRE2 server for automated modelling [24]. Only one NLR NACHT structure, the 4KXF structure of NLRC4 [25], is available in the PDB at present and this was used as a template. Inspection of the resulting alignment and model showed that while the NBD, HD1 and WH domains (217–632) were clearly homologous between NOD2 and NLRC4, the rest of the exon, which forms HD2 and part of the LRR domain in NLRC4, showed a more ambiguous alignment and so was not considered further. The final model represents NOD2 residues A217–C632.

Homology models of the CARD of murine CARD9 and human CARD9 from residue 6–100 were built using the CARD from CARD11 (PDB 1D: 4I16) [26] as a template. Models were generated using the model-default script from the MODELLER package v9.12 (http://salilab.org/modeller/).

The amino acid sequences of full length murine CARD9 (GENBANK: NP_001032836.1) and human CARD9 (GENBANK: NP_43470.2) were aligned and the percentage identity and similarity calculated using Clustal Omega [27].

## 3 Results

### 3.1 There is no interaction between the CARDs of NOD2 and CARD9

CARD-mediated protein–protein interactions play an important role in numerous immune signalling pathways such as RIG-1 mediated viral sensing, inflammasome formation and facilitating NOD1/2-mediated NF-κB signalling via RIP2 [28], [29], [30]. NOD2 and CARD9, which both possess CARDs, work synergistically to drive p38 and JNK signalling following activation of NOD2 and have been shown to interact [17]. Currently nothing is known about how this interaction is mediated, although theoretical models have suggested the involvement of CARD:CARD interactions [31].

To test whether the NOD2 CARD9 interaction is CARD-mediated we expressed the CARDs of human NOD2, murine CARD9 and human RIP2 as recombinant proteins fused to solubility-enhancement and epitope tags. Interactions were assessed using a modified version of the protocol established to study interactions between the CARDs of NOD2 and RIP2 [22]. Specifically, bacterial cultures containing overexpressed recombinant protein were resuspended and combined prior to sonication. Amylose resin based affinity chromatography was used to purify proteins fused to MBP and co-purifying interacting proteins were detected by Coomassie Brilliant Blue staining following SDS–PAGE.

**Fig. 1:**
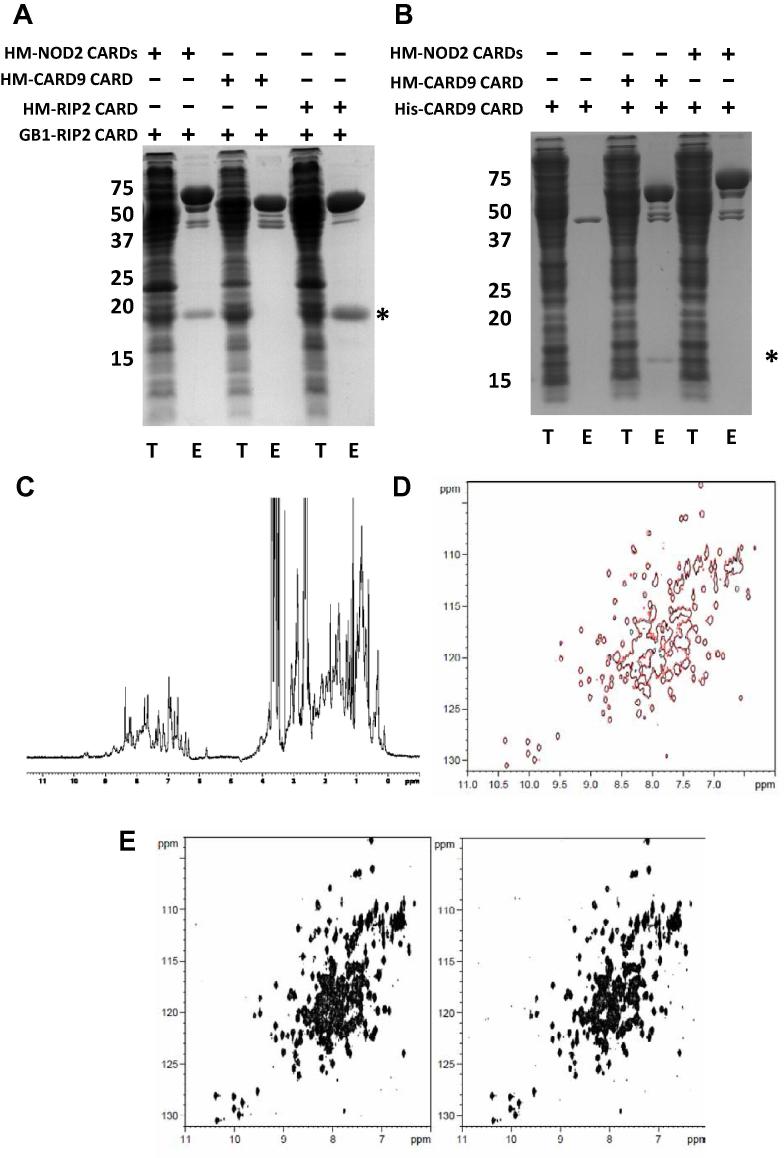
The CARDs of NOD2 and CARD9 do not interact. His-MBP tagged NOD2 CARDs, CARD9 CARD and RIP2 CARD were used to co-purify GB1-RIP2 CARD (A) or His-CARD9 CARD (B). The CARD9 CARD did not interact with either the CARDs of NOD2 or RIP2, but did display homomeric interactions. The asterisks represent the location of the GB1-RIP2 CARD (A) and the His-CARD9 CARD (B). T = total cell lysate; E = Elution post amlyose affinity purification. (C) 1D (^1^H) NMR spectrum of CARD9 confirms the protein is tertiary structured. (D) Overlay of 2D (^1^H/^15^N) HSQC spectra of NOD2 (28–218) in the absence (red; 1:0 equivalents) and presence (black; 1:1 equivalents) of CARD9 CARD. (E) Side-by-side 2D (^1^H/^15^N) HSQC spectra of NOD2 (28–218) in the absence (left) and presence (right; 1:1 equivalents) of CARD9 CARD.

Consistent with previous work the NOD2 CARDs successfully pulled down the RIP2 CARD, which was also capable of mediating homomeric RIP2 interactions (Fig. 1A). However, we detected no interaction between the CARD of CARD9 and either the NOD2 tandem CARDs or the RIP2 CARD; although the CARD9 CARD could facilitate weak self-association as has been previously reported [32] (Fig. 1A and B). The inability of the tandem CARDs of NOD2 and the CARD9 CARD to interact was further confirmed using NMR chemical shift studies. 1D (^1^H) NMR spectrum confirmed that the CARD of murine CARD9 is tertiary structured as shown by the ring-current shifted methyl signals (<0.5 ppm) resulting from formation of a hydrophobic core (Fig. 1C). ^15^N-labelled NOD2 (28–218) is also tertiary structured, displaying a generally good dispersion of peaks in the 2D (^1^H/^15^N) HSQC spectra and chemical shift values >9 ppm (due to hydrogen bond formation; Fig. 1D and E). However, there are no significant chemical shift changes in the presence of equimolar CARD9 CARD indicating that there is no interaction between the two recombinant proteins under the conditions studied (Fig. 1D and E (right panel)). Together these approaches conclusively support the view that the CARDs of NOD2 and CARD9 do not directly interact.

Comparing the sequences of murine CARD9 and human CARD9 indicated that these proteins are 86% identical and 91% similar across their entire sequence, both identity and similarity increasing to 95% for the CARD (Supplementary figure 1A). Homology models of the two CARDs confirmed that four of the five substitutions were in helix 1 and the fifth in helix 6. None of the mutations affect the overall protein fold or the electrostatic properties of the CARDs (Supplementary Fig. 1B and C). This is consistent with the conserved biological function of the molecules and allows us to conclude that it is highly unlikely that the CARDs of human CARD9 and human NOD2 would interact either.

### 3.2 The NACHT and CARD–NACHT linker of NOD2 are important for interaction with CARD9

**Fig. 2:**
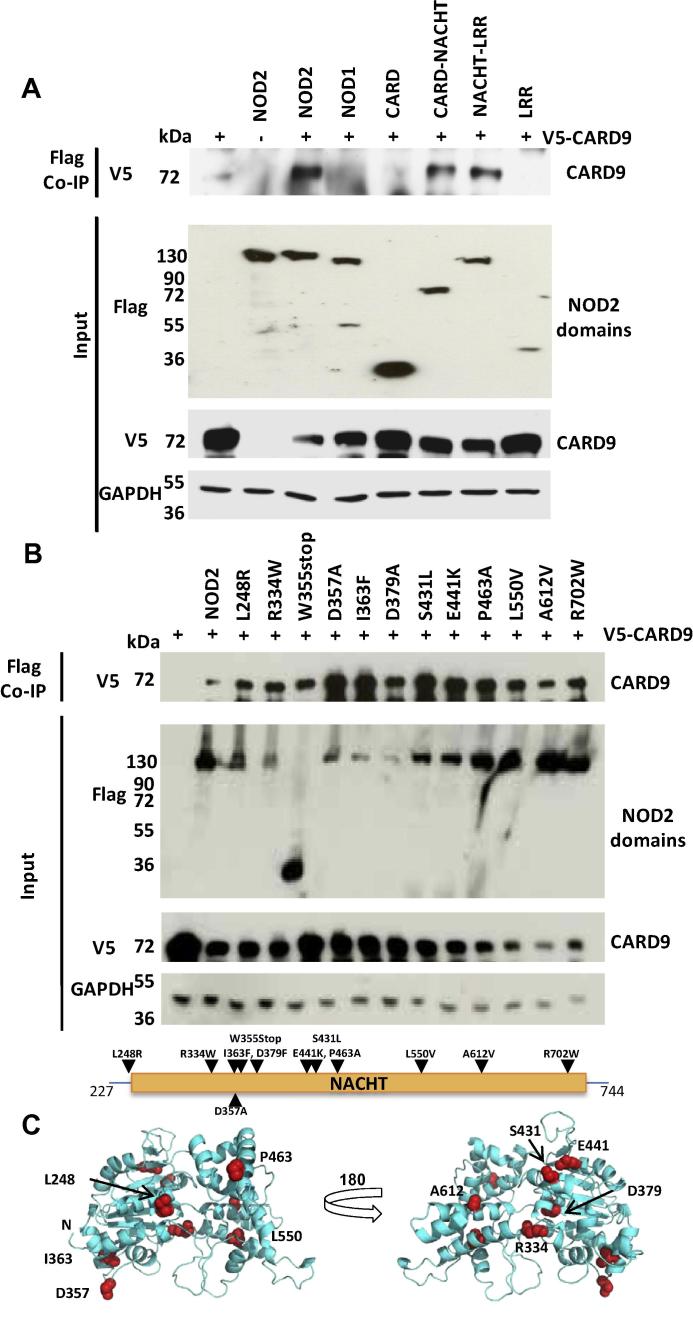
NOD2 interacts with CARD9. (A) HEK293T cells were transiently transfected with V5-CARD9 and FLAG-NOD2 full-length and domain truncation expression constructs; or with (B) V5-CARD9 and FLAG-NOD2 NACHT polymorphism containing constructs. 24 h later cell lysates were subjected to co-immunoprecipitation using anti-FLAG antibody and samples analysed by Western-blotting. Neither the CARDs alone, nor the LRRs alone interacted with CARD9; and none of the polymorphisms disrupted the interaction. The relative position of the polymorphisms is shown on a schematic of NOD2. (C) Location of the NOD2 SNPs on a homology model of the NOD2 NACHT. SNPs are coloured red and the side chains shown as spheres. Images were generated using PYMOL (Schrödinger).

Given the inability of the respective CARDs to interact we used domain truncations of FLAG-tagged NOD2 to map the region required for interaction with CARD9. HEK293 cells were transiently transfected with NOD2 truncations and V5-tagged CARD9 and proteins immunoprecipitated after 24 h. CARD9 was immunoprecipitated by full-length, CARD–NACHT, and NACHT–LRR NOD2 constructs (Fig. 2A). Neither the CARDs nor the LRRs alone interacted with CARD9, suggesting that the NACHT domain of NOD2 has a critical role in mediating interaction with CARD9.

**Fig. 3:**
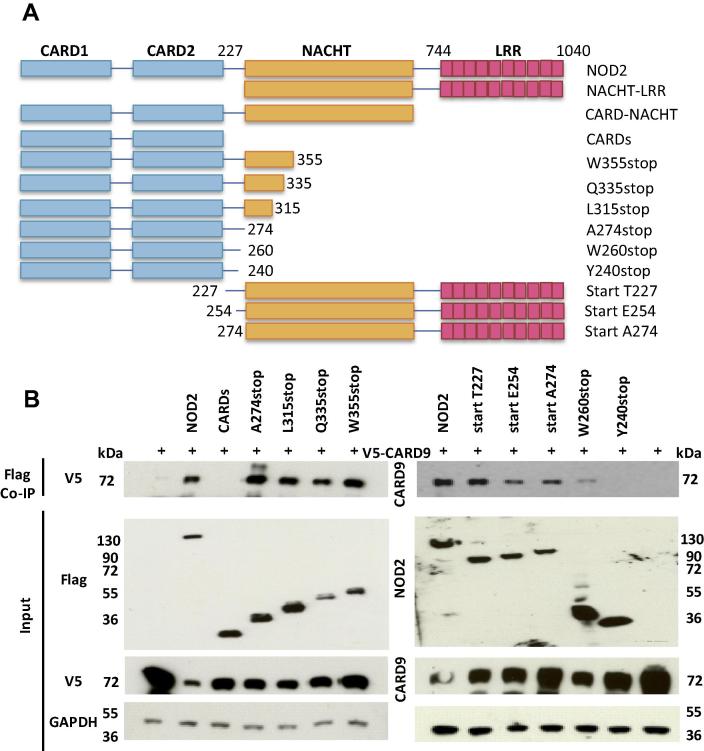
(A) Schematic representation of the NOD2 deletion constructs. (B) HEK293T cells were transiently transfected with truncated FLAG-NOD2 expression constructs and V5-CARD9 and co-immunoprecipitated after 24 h using anti-FLAG antibody and samples analysed by Western-blotting. Inclusion of the NOD2 CARD–NACHT linker, or the NACHT domain itself, facilitated interaction with CARD9.

To further investigate the importance of the NOD2 NACHT domain we tested the impact of a panel of Crohn’s Disease associated single nucleotide polymorphisms (SNP) [33], [34], [35], [36] spanning the NACHT on the interaction with CARD9. This included the widely studied R702W SNP. We also tested the hyperactive Blau Syndrome associated SNP R334W [37]. All of the polymorphisms still interacted with CARD9 indicating that these residues, and potentially the surrounding regions of the NACHT, were not crucial for CARD9 interaction (Fig. 2B and C). It also demonstrates that the clinical impact of the R334W and R702W SNPs is unlikely to result from alteration of the CARD9 and NOD2 interaction. Interestingly, the W355stop SNP, which lacks any sequence downstream of this tryptophan, still immunoprecipitated Card9 suggesting that a region between the NOD2 CARDs and W355 could mediate interaction. Consequently, we generated further C-terminal NOD2 truncations (Fig. 3A) and tested their interaction with CARD9. All of these truncations, including A274stop which lacks any of the NACHT, retained the ability to interact with CARD9 (Fig. 3B). Together with the failure of the CARD only construct (residues 1–227; Figs. 2A and 3B) to interact with CARD9 this indicated that residues 228–274 contain a critical region for CARD9 interaction.

To characterise the role of the CARD–NACHT linker in more detail we expanded our range of mutants to alter the amount of linker present (Fig. 3A). Extension of the C-terminus of the CARD only construct to residue 240 did not result in interaction with CARD9 (Fig. 3B). However, addition of a further twenty residues, to position 260, resulted in CARD9 being immunoprecipitated, albeit more weakly than with the the wild-type protein, or other truncations (Fig. 3B). All of our N-terminal truncations were able to immunoprecipitate CARD9, including a construct beginning at 274 which therefore lacks the CARD–NACHT linker (Fig. 3B). Intriguingly both the constructs 1–274 and 274–1040 interacted with Card9 thereby implying the presence of at least two binding sites in NOD2 for CARD9; one located in the CARD–NACHT linker (between residues 241 and 274) and the other in the NACHT domain.

Together these results support an interaction between NOD2 and CARD9. This interaction is not mediated by the CARD domains, but instead involves two regions in NOD2 – one linking the CARDs and NACHT, the other within the NACHT itself.

## 4 Discussion

The pattern recognition receptors NOD1 and NOD2 play an important role in the pro-inflammatory immune response to bacterial infections. Their ability to activate NF-κB-mediated transcription following interaction with RIP2 has been widely studied. Our understanding of how these receptors activate alternative signalling pathways, such as those utilising p38 and JNK, is limited. However, it has been previously shown in mice that engagement of the adaptor protein CARD9 is crucial in facilitating NOD2-initiated p38 and JNK signalling, with the two proteins working in synergy to mediate this response [17]. How this is achieved, and how NOD2 and CARD9 interact has not previously been understood. In this work we have identified two regions of NOD2 capable of binding CARD9; one in the linker connecting the CARDs and the NACHT, and one in the NACHT itself. These binding surfaces may by spatially adjacent to one another in the tertiary structure of the protein. A random mutagenesis study of NOD2 identified two residues, A232 and V256, in the linker region that when mutated resulted in a significant loss of NF-κB signalling [38]. Whilst for A232 this correlated with a loss in protein expression this was not the case for V256, suggesting that the linker may be more important for NOD2 functionality than previously assumed. Somewhat surprisingly and despite the known importance of CARDs in mediating protein–protein interaction in immune signalling complexes we saw no evidence that the CARDs of NOD2 and CARD9 could interact with one another.

In this study we used murine CARD9 and human NOD2. In light of the conserved biological function [15], [16]; the high level of sequence identity (Supplementary Fig. 1A); and the conserved nature of the electrostatic surfaces (Supplementary Fig. 1C) between the human and murine proteins we are confident that our observations and conclusions remain valid for interactions between human CARD9 and NOD2.

Our observations raise interesting questions about the molecular composition and formation of the NOD2:CARD9 signalling complex. The lack of NOD2 CARD9 CARD-mediated interaction suggests that NOD2 could interact concurrently with RIP2, via its CARDs, and with CARD9 via its linker/NACHT. This would enable simultaneous activation of NF-κB, p38 and JNK signalling pathways from the same macromolecular complex. Hsu and colleagues reported that the presence of RIP2 enhances the interaction between NOD2 and CARD9 [17].This could be through promotion of conformational changes due to receptor activation which expose the CARD9 binding motifs on NOD2; or by promoting protein clustering and thereby increasing the avidity of the NOD2:CARD9 interaction. CARD9 contains a predicted coiled-coil domain (CCD). CCDs are widely reported to mediate protein–protein interactions and it remains entirely feasible that the CCD mediates interaction with NOD2. However, difficulties in expressing the CCD independently of the CARD [32] have made this hypothesis impractical to test to date.

In conclusion, we have identified regions of NOD2 important for CARD9 interaction. These now require functional interrogation to determine precisely how the two proteins interact and whether these regions could serve as modulators of receptor signalling with the potential for the regulation of a range of signalling pathways.

## Appendix A Supplementary data

Supplementary data 1 Murine CARD9 and Human CARD9 are highly similar. (A) Clustal Omega generated sequence alignment of murine CARD9 (top) and human CARD9 (bottom). The consensus sequence is shown underneath. The CARD is delineated by a solid black bar and the residues forming the hydrophobic core of the CARD are highlighted in grey. (B) Homology models of the CARD from human CARD9 (blue) and murine CARD9 (brick red). Residues that differ between the two CARDs are shown in stick representation and the models are overlayed to emphasise their similarity. (C) Electrostatic surface of the human (left) and murine (right) proteins. The orientation of the electrostatic surface is identical to that presented in panel (B).
