# Supplementary figures and images for: Interaction between NOD2 and CARD9 involves the NOD2 NACHT and the linker region between the NOD2 CARDs and NACHT domain
Source: FEBS Lett. 2014 Aug 25;588(17):2830–6. doi: 10.1016/j.febslet.2014.06.035 (PMC4158416; doi:10.1016/j.febslet.2014.06.035)

## Slide 1
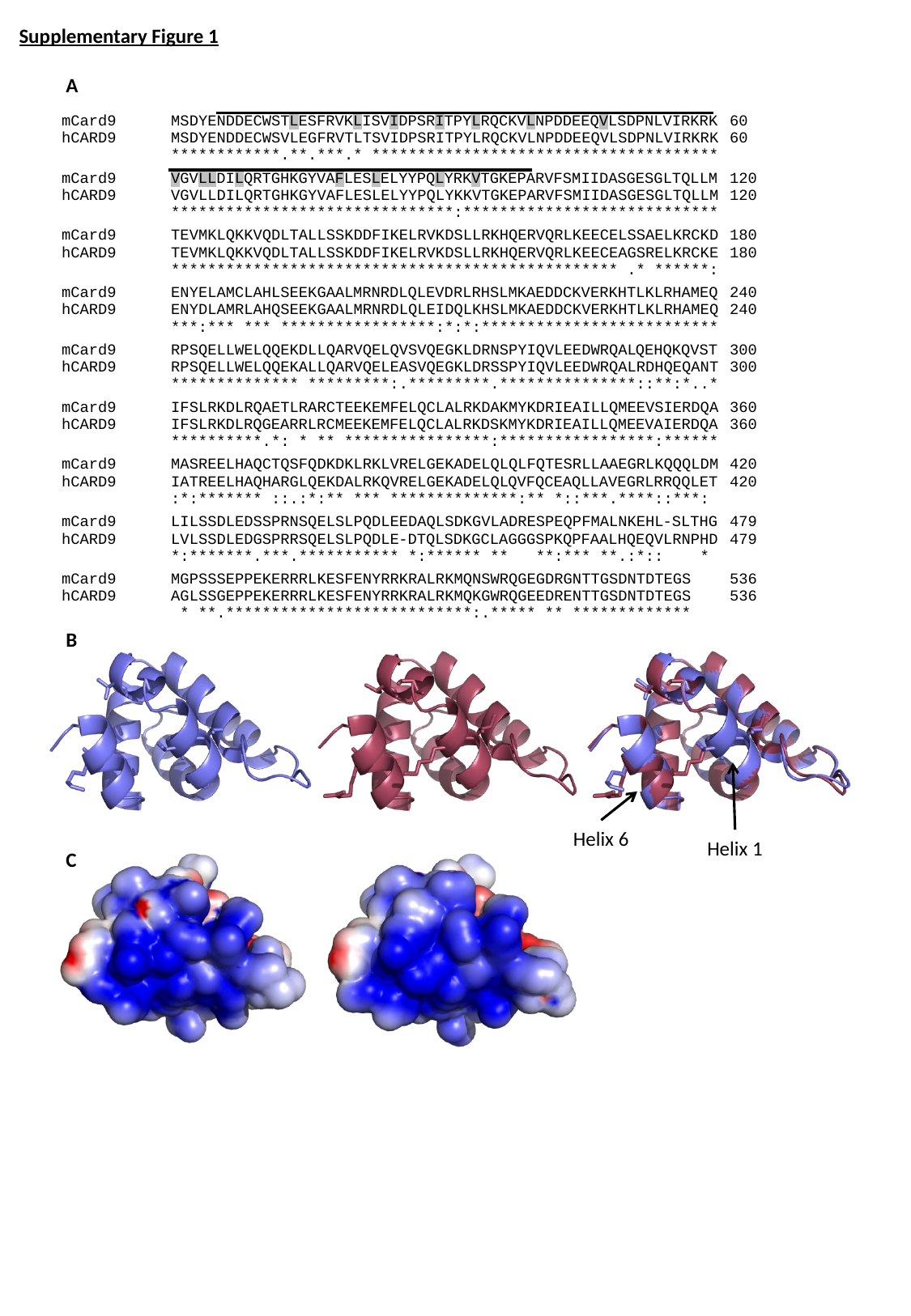

Supplementary Figure 1
A
B
Helix 6
Helix 1
C

Supplement: Supplementary data 1 — Murine CARD9 and Human CARD9 are highly similar. (A) Clustal Omega generated sequence alignment of murine CARD9 (top) and human CARD9 (bottom). The consensus sequence is shown underneath. The CARD is delineated by a solid black bar and the residues forming the hydrophobic core of the CARD are highlighted in grey. (B) Homology models of the CARD from human CARD9 (blue) and murine CARD9 (brick red). Residues that differ between the two CARDs are shown in stick representation and the models are overlayed to emphasise their similarity. (C) Electrostatic surface of the human (left) and murine (right) proteins. The orientation of the electrostatic surface is identical to that presented in panel (B). [file mmc1.pptx]
